# It’s a Mindset Revolution! Co-creating inclusive spaces of participation on youth mental health

**DOI:** 10.1177/14767503251320616

**Published:** 2025-02-17

**Authors:** Sonia Bussu, Katy Rubin, Niamh Carroll, Zarah Eve

**Affiliations:** 1724 University of Birmingham, UK; The People Act; 42nd Street; 5289 Manchester Metropolitan University, UK

**Keywords:** ART, Participatory democracy, youth participation, legislative theatre, assemblage theory

## Abstract

The Mindset Revolution project aimed to strengthen youth voice on mental health policy and practice. We worked with a diverse group of young people to co-create digital and in-person spaces of participatory democracy and action research. We combined play, art and creativity to challenge power dynamics and name structural racism, sexism, ageism, classism, and ableism as forces in institutional practice in the mental health system. Our approach reflected the participatory ethos and ambition for social transformation underpinned by Action Research with a concern for Transformations (ART). By taking an assemblage perspective, the paper highlights the relational and situated nature of participatory democracy and action research, warning against top-down designs and advocating for context-specific and emergent practices that can foster sustainable social change. The paper emphasises the complexity of achieving policy impact, highlighting barriers such as limited space and time for trust-building processes, limited resources, and stringent funding requirements. It advocates for a flexible, long-term approach that integrates diverse, intersectional and radically inclusive spaces of participation, embedded in the community that can take ownership beyond small-scale projects. Transformative change in youth mental health can only occur when it is co-created with and owned by young people and their communities.

## Introduction

Young people are often categorised by adults as missing or marginalised actors within public participation, requiring “reaching out”. In reality, we know that they perform democratic actions on multiple levels, as citizens, activists and in everyday settings that help revitalise public life from the grassroots (Nishiyama, 2017, p. 7). Although young people tend to engage less in more institutionalised spaces of participation, showing declining interest in representative democracy, elections and political parties (Grasso et al., 2018), the literature tells us that they can and want to play a role in decision-making when offered the opportunity (Austin, 2010). The Mindset Revolution (MR) project^1^ aimed to support young people to co-create and shape their own spaces of democratic participation and build a collective voice for change on youth mental health policy and practice in Greater Manchester, UK.

Rather than confining democracy to institutional arrangements, we understand it as participatory (Pateman, 1970) and a way of life (Dewey, 1927). Democracy as everyday practice is relational and situated, realised through multiple, overlapping and interconnected spaces (Bussu et al., forthcoming). Following Massey (2005), space is imbued with relationships and power struggles. If time is the dimension of succession, where things happen sequentially, space is the dimension of simultaneity and multiplicity, opening alternative possibilities to the status quo. This conceptualization of democratic space informed our practice and epistemology, encouraging plural experimentation and co-creation with young people, to prefigure novel, youth-led understanding of both mental health and democratic participation. Unlike traditional approaches to youth democracy that tend to favour adult-led designs mimicking adult spaces (e.g., Youth Parliaments), MR started from the young participants’ perspectives, experiences and capabilities to participate to create conditions for relational change within spaces of which they had ownership. We bridged participatory democracy and action research of Freirean tradition (Freire, 1968) to flip conventional public engagement. This approach supported young people to co-create their participatory process on mental health, invite policymakers to engage with their agenda, and evaluate their own impact.

This work was guided by two main research questions: How are spaces of youth participatory democracy transformed when young people shape and evaluate these spaces through creative and reflective practice? How can researchers and practitioners support meaningful youth participation to enable relational change and embed social change? We recruited 23 young people (16–25 years old) through a local university and local youth organisations who were partners in the project. We co-created three overlapping spaces of participation. Firstly, Legislative Theatre (LT) (Boal, 1998) enabled the young people to represent their own experience of intersectional exclusions within the mental health system, interrogating and challenging assumed expert knowledge that continuously silences and invalidates their perspective. The use of participatory art can encourage political experiences where individuals feel personal attachments to society, strengthening their commitment to democratic change (Thévenot, 2014, p. 149). Secondly, a digital participatory platform allowed young participants to autonomously engage in digital collaborations to create, share and discuss resources on youth mental health. Finally, the young people used creative research methods to explore their experience as participants and co-creators and evaluate both the process and their social impact.

To develop an analysis that could capture the complexity and playful messiness of MR’s multiplicity of participatory spaces, this paper employs the concept of assemblage. Assemblage theory (DeLanda, 2016; Deleuze & Guattari, 1988) understands reality as composed of various human and non-human (e.g., material resources, physical space; technology) elements that interact and coalesce to form temporary and ever-changing configurations called “assemblages” (Bussu et al., forthcoming). Assemblage helps us look at how different participatory spaces, and the (power) relationships they are imbued with, coexist, interact and change. It recognises participants as dynamic actors and provides a lens to trace how their identities emerge and are continuously redefined through participation; how they are embedded within existing relational structures; and their abilities to form new connections.

Action Research with a concern for Transformations (ART) was an important component of this participatory assemblage to foster epistemic justice, by mobilising social learning grounded in the young participants’ experience and conscientisation process (Freire, 1968). Bradbury (2024) advocates for action research as a transformative methodology that reconnects knowing and doing and promotes relational dynamics to address real-world issues. It fosters transformative change through experiential learning, reflection, and collaborative action (Bradbury et al., 2019). To open space for youth-led change and possibilities for transformative relational dynamics means giving up on preconceived notions of what participation and research mean and do, as well as of linear implementation and impact. This entails flexibility and adaptability. Inevitably we navigated several barriers, and our assemblage-informed analysis provides a novel perspective on the challenge of co-creating and embedding social change.

The next section brings into dialogue assemblage theory, youth participation and action research to make sense of the complex interactions within this project. The following section presents our project on youth participation on mental health. We then discuss findings through an assemblage lens and finally offer some concluding remarks on the potential and limitations of the approach, highlighting the paper’s contribution to action research with young people for social transformation.

## Youth participation through an assemblage lens

Youth agency entails a plurality of notions of participation and activism (Börner et al., 2021), which also questions the boundaries between activism and everyday lives to capture “day-to-day practices” (Horton & Kraftl, 2009, p. 16) and “implicit activisms” (Horton & Kraftl, 2009, p. 17). In MR, participation emerged through interacting spaces and practices, online and in person, to adapt to the participants’ different circumstances and resources and their experience of intersectional exclusions. Bustamante Duarte et al. (2018) explored the benefits of combining different participatory approaches when working with young migrants during resettlement. When using different spaces in tandem in the same project, with each different approach filling in the gaps left by the others, we can create a richer environment for discovery and reflective enquiry (on a similar approach, see Bowler et al., 2021).

MR enabled multiple opportunities for engagement, expanding existing practice of action research with youth for social change (Flicker et al., 2008; Luguetti et al., 2024). An approach grounded in flexibility and adaptability translated into different avenues for participation, from light participation (e.g., sporadic participation in youth-led surveys and opportunities to submit policy proposals or create new content for the digital platform) to medium participation (e.g., participation in working groups on research and design activities) to heavy participation (e.g., participation within a core group of legislative theatre young facilitators that met regularly to plan and deliver sessions with their peers) (on this also see Allahwala & Bhatia, 2022). Even within the “heavy” levels of participation the same flexible approach applied. The young people were able to lean on each other and if one of them needed to take a back seat at a given session, the group learned how to adapt and step in. These spaces were youth-led, and by this we mean that each group of young people chose how to shape the spaces opened by the adult partners (e.g., legislative theatre; digital platform; participatory evaluation), making decisions on which issues to explore and how. Furthermore, the young people facilitated these spaces with help from the adults in the team, who ensured organisational and logistical support throughout, as well as acting as brokers with local institutions and mental health services on behalf of the young people.

However, as detailed in the analysis section, we acknowledged subtle power hierarchies that can risk turning young people’s participation into compliance with adult ideals and can challenge the ambition for horizontal relations and exchanges on equal terms (Lundy & O’Donnell, 2021). As academics and practitioners, we recognised our life experiences were often very different from those of the young people we worked with. Mindful of risks of extractive practice, we tried to create space for subversive discussions that enabled the young people and the adults to reflect on positionality and question adult biases and assumptions about youth engagement and youth mental health. This process over time helped us foster more authentic collaborations as a precondition for transformational capacities (Luguetti et al., 2024).

In the analysis that follows, we present MR as a participatory assemblage, where different human and non-human components come together to shape mutual learning for transformative change. The assemblage lens helps us pay attention to the plurality of spaces and interactions that allows for the expression of variable forms of “experiencing, belonging and acting” (Kallio et al., 2015, p. 113). Following Börner et al. (2021, p. 278), we understand agency as capturing these “diverse facets of young people’s engagement, their emotional relationships, as well as their (changing) interactions with their everyday environments”. It is the sense of ‘belonging’ and ‘connection’ that proved crucial for young people to start perceiving themselves as part of the solution. Relational, conceptual and experimental spaces combined to foster “knowledge creation as social practice” (Bradbury, 2022, p. 5).

Assemblage epistemology emphasises the dynamic and relational nature of participatory spaces, highlighting how they evolve in response to contextual factors (McFarlane, 2011a). The impact of participatory processes emerges from the interactions among various actors, events, materials, and processes, and their *labour of (dis)assembling* across complex social and political spaces (Massey, 2005; Shore & Wright, 1997). This pragmatic approach serves as a valuable methodological tool for understanding the diverse elements that drive participatory ideas and approaches.

To operationalise these epistemological commitments an ethnographic sensibility is required that allows for fine-grained tracing of sites and situations (Herbert, 2000, p. 551). Through creative research methods and participants observations, we iteratively traced interactions within the assemblage shaped by: (a) discourses and spaces of youth-led participation (i.e., Legislative Theatre play, digital participation process, community partners’ activities on youth-mental health); (b) technologies of power of local institutions (e.g., policymaking actors, academia); and (c) “scientific” and “adult” modes of knowledge production and conceptualisations of youth mental health. Inevitably, this participatory assemblage was structured by forms of power, capital, and dominant discourses, but it also exceeded these structures and contained within itself the capacities for becoming something different (McFarlane, 2011b).

## Case study: Youth participation on mental health in Greater Manchester

MR was a partnership between academics, practitioners and third sector and community groups working with young people. We employed a diverse group of 23 16–25-year-olds as co-creators and co-researchers to explore what happens when participatory spaces are designed with participants, who reframe the problems, co-develop the agenda and reinvent interactions with the state to influence policies. A recruitment ad was shared through local partners with youth groups and their networks. Students from one local university were also invited to apply for internships linked to the project. The selection process, based on a short application form to gauge interest and motivation to participate, ensured diversity along gender, ethnicity, and age. Most participants were from less privileged socio-economic backgrounds, and all had experience of mental health challenges, either directly or through family members. While some were already active in local youth organisations, the majority had limited or no prior experience of political engagement and/ or grassroots action.

The adult partners^2^ had already worked together as part of a three-month project, Optimistic Minds, a Legislative Theatre (LT) process involving seven young people to rethink conceptualisations of evidence-based policymaking on mental health. LT (Boal, 1998) is an established participatory approach for creative, community-led policy change. In LT, audiences and policymakers watch a play based on the participants’ experiences of oppressive policies and practices (e.g., in mental health provision). Then, audiences act onstage to rehearse ways to confront the problems presented. Based on these improvisations, actors and audiences propose ideas for new laws, rules and policies to address the problems, working together with advocates, organisers, and government representatives. Finally, everyone present votes to prioritise the new rules, and policymakers commit to measurable actions to move forward the proposals.

**Figure 1.**
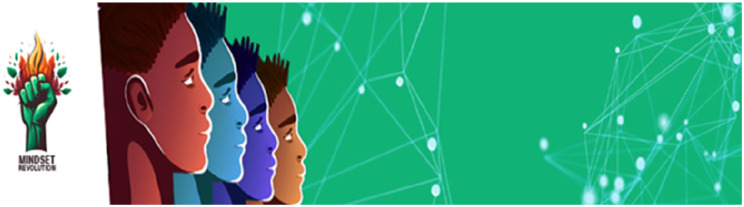
Logo of mindset revolution designed by the young people

**Figure 2.**
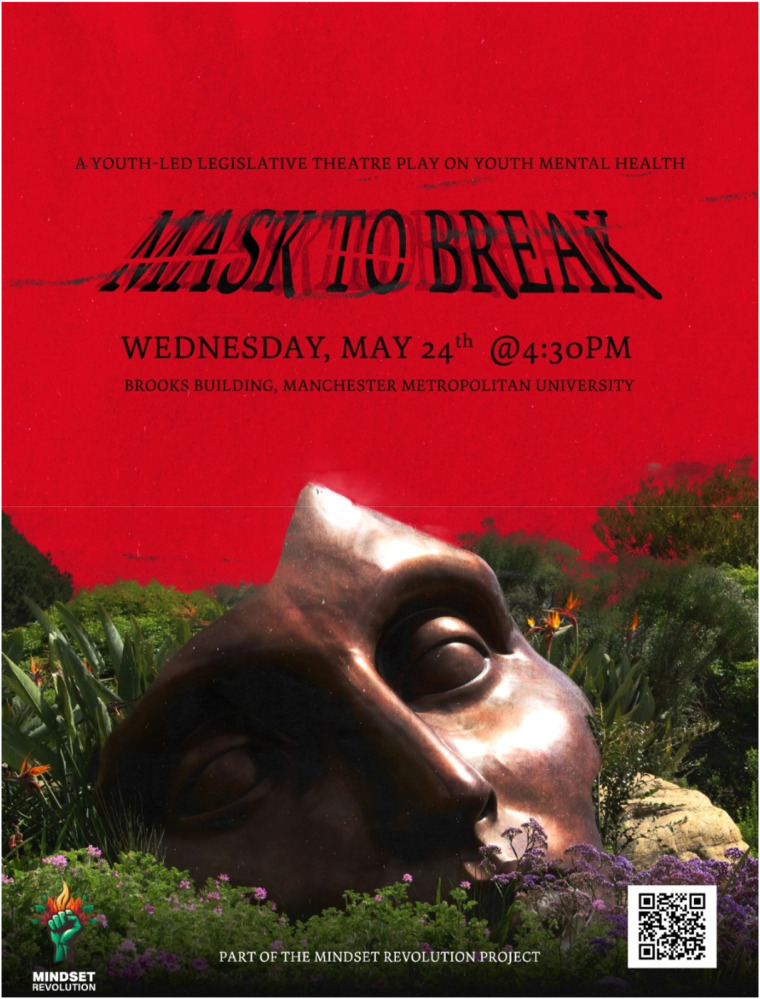
Poster of mindset revolution’s youth-led legislative theatre play, mask to Break, by digital peer designers

In MR, the young people, who were paid for all their work in the project, joined three core groups, based on their interests (see logo designed by the young people in Figure 1). One group used LT to continue to explore barriers to mental health support, building on the Optimistic Minds project. Four young people who had previously been involved in Optimistic Minds were trained by an expert LT practitioner (Rubin) and facilitated their own LT process involving six new young people. The play they produced, *Mask to Break* (see poster in Figure 2), represented the complex journey of a Global Majority young person who tries to navigate a mental health system entrenched in ableist, classist and racist practices and behaviours, and which is incapable of responding to the different needs of diverse cultures.

**Figure 3.**
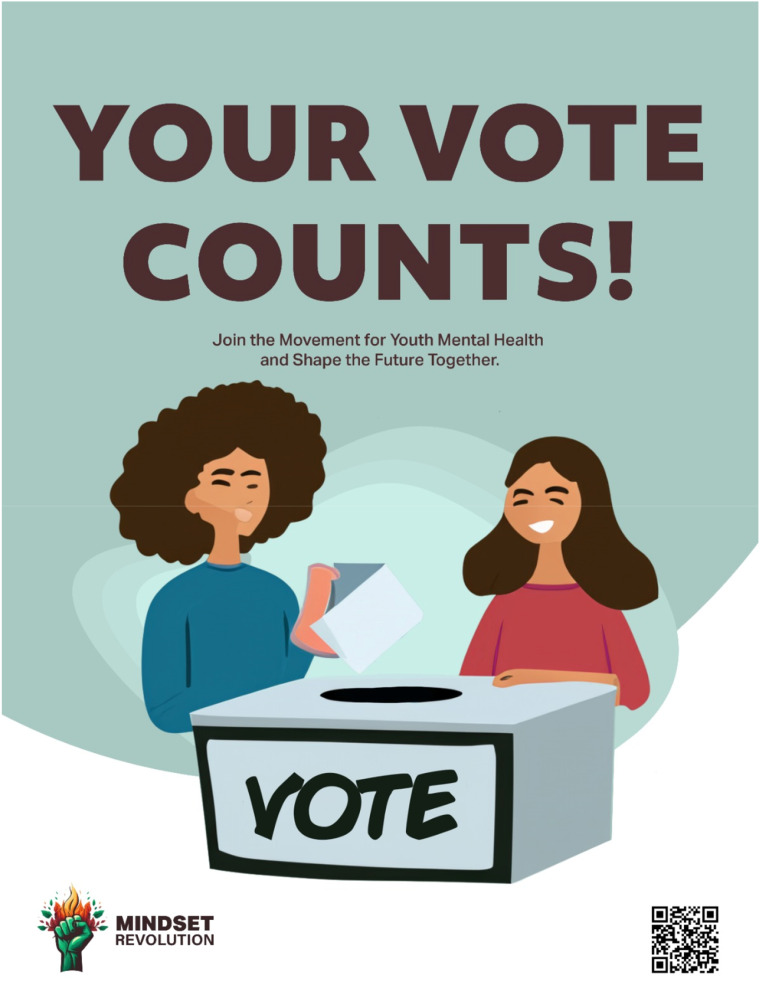
Flyer for the youth-led digital process by digital peer designers

Another group, with support from a team of civic tech developers, designed and ran a digital participatory process on Decidim, an accessible digital platform for citizen participation (Bussu et al., 2024). The digital process aimed to reach out to wider youth in the region to (a) map out problems with mental health support from young people’s perspective; (b) invite proposals to address these problems; (c) discuss and develop the proposals with peers and policymakers through a series of youth-led online dialogues; (d) engage relevant services and policymakers to identify feasibility issues. Figure 3 shows the flyer to invite voting on proposals.

This group of six digital co-creators, with support and contributions from youth within and beyond the project, also developed an online space for young people to share resources promoting mental health. The LT and the digital processes informed 12 policy ideas, which placed emphasis on the need for a stronger intersectional approach to mental health support.^3^ Table 1 details some of the policy proposals that emerged throughout the project and that informed a policy brief submitted to the UK Parliament.

**Table 1.** Mindset revolution policy recommendations for intersectional mental health support.

Cultural competency quality assessment framework	Establish a cultural competency quality assessment framework and promote Diversity Experience Days and cultural competency training for all mental health professionals. The training and framework should be developed alongside young people, relevant communities and organisations to a) establish formal standards and guidelines for working with various diverse backgrounds (culture, gender, class, etc.) and b) give organisations a stamp to demonstrate their competency.
Young people’s advocates	Increase levels of support to help young people navigate the mental health system, through advocates with understanding of the young person’s background.
Youth-designed mental health spaces	Ensure youth mental health spaces in both educational and NHS are designed by young people from different backgrounds, with an accountability process that ensures the end design of these spaces accurately reflects input from young people.
Mental health training in education settings	Make it compulsory for all educational staff members in schools, colleges, and universities to be trained in mental health to increase early intervention and improve respect for those experiencing mental health issues.
Coproduction with young people	Promote co-production with young people at the forefront: School staff members, medical and support service providers, parents and charities should be able to collaborate in a positive way with young people to facilitate access to appropriate mental health support.

**Figure 4.**
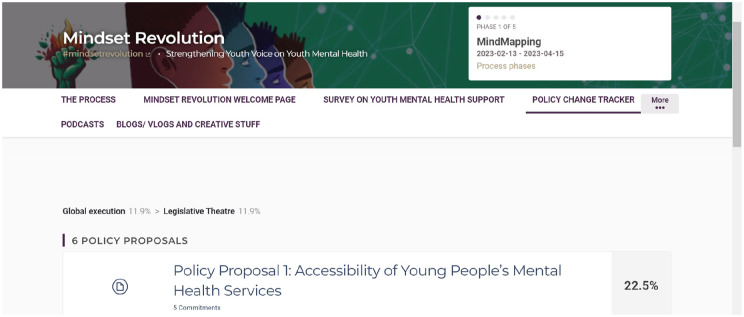
Accountability function on the mindset revolution platform

A crucial aspect of our approach was an emphasis on “participatory scrutiny”; the Decidim platform includes an accountability function to monitor progress on implementation, which the young people renamed “policy change tracker” (Figure 4). Accountability became a highly visible process, with actions clearly linked to organisations that had made specific commitments (Bussu et al., 2024). Our ambition was that this focus on participatory scrutiny could empower young people, with support from community partners, to demand follow-up on decisions and explore barriers to implementation in a dialogue with policymakers and services. The risk is that seeing limited progress over time also generates frustration among young participants.

To evaluate the approach, seven peer researchers conducted a participatory and developmental evaluation (Percy-Smith et al., 2019), supported by academics. They used creative methods, such as reflective journals, poetry, and a podcast series, alongside traditional methods (e.g., interviews and focus groups with peers and partners) reclaimed as youth-led practice. They produced an evaluation playbook^4^ detailing their learning and assessing their experience and perceived impact. The following sections will explore how these methods contributed to new insights on mental health and youth participation. As nicely put by Ritterbusch et al. (2020, p. 5):

[The peer-led] process reframes the research environment as a space for collective reflection on the past, present and future and creates contexts for self-empowerment where young people recognise the strength implicit in their survival.

**Figure 5.**
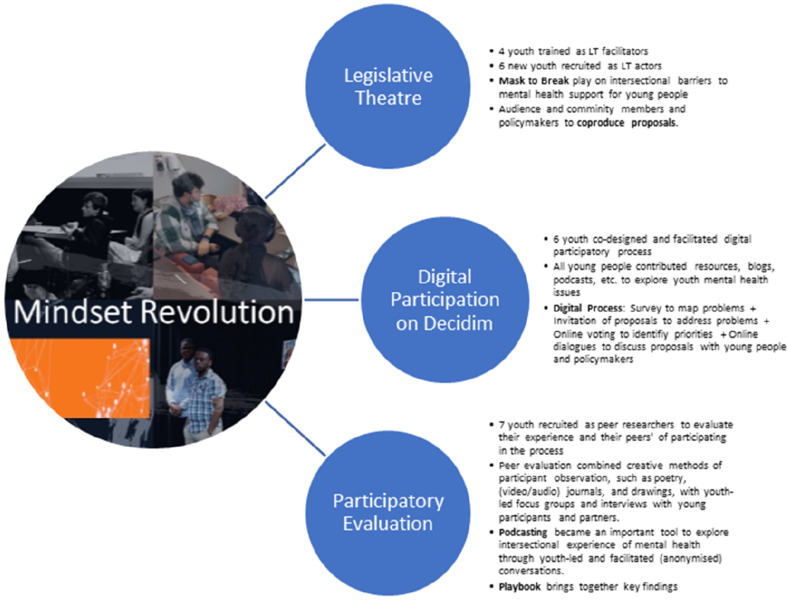
Mindset revolution’s multiple participatory spaces and practices

Emergent findings were integrated into a flexible process design, with weekly reflective meetings deepening understanding of personal stories and structural challenges. Project activities aimed to link and build on community partners’ work to embed impact beyond short term funding, though this proved challenging, as discussed further in the following sections. Figure 5 summarise the different participatory spaces and activities.

As well as securing ethics approval from both academic partners, the team ensured that the safety protocols routinely used by partnering youth organisations were applied to our project activities, implementing risk assessments and providing access to mental health support when needed. The partnership also co-developed a safety protocol for online engagement and strengthened moderation functions to support online safe spaces to debate sensitive issues. This also entailed setting up alerts to flag posts requiring attention. Reflective sessions enabled young people to share their experiences with mental health and their participation in the project. This helped adult partners tailor support and communication better, as awareness of the group’s diverse needs and skills deepened. Monthly meetings with all partner organisations, and more regular planning sessions involving academics and practitioners working directly with the young people, aimed to support reflection on ethical practice and the everyday ethical issues that arise in the doing of research.

## Assembling spaces of youth participation

This section uses an assemblage perspective to reveal the multiple and interrelated material and social relations, cultures, and ecologies (McFarlane, 2011a) that produce and shape a participatory process for social change. Findings are based on the participatory evaluation of this project, which includes over 100 hours of participatory observations using a range of creative methods (e.g., drawings, pictures, poetry) at 90 in-person and online weekly meetings and rehearsals; four youth-led in-person focus groups with participants; five one-to-one and group interviews with partners carried out on Zoom, and two coproduction workshops to reflect on data collection and interpretation with the peer researchers. We present the findings with a focus on relationality, interactions and power dynamics to reflect on the transformative ambitions of these youth-led spaces of participation and the barriers they navigated. We discuss how MR created conditions for relational change and explore the ongoing labour of (dis)assembling that underpins (dis)embeddedness of youth participation for social change.

### Creating conditions for relational change

An assemblage lens places emphasis on dynamic networks and interactions, emphasising the fluid and contingent nature of socio-material realities that create possibilities of change. Young people’s participation often depends on socio-economic factors and personal circumstances but also, as noted by McFarlane (2011b), a particular atmosphere of reception in different groups, the materiality of the documents and technologies we use, as well as serendipitous moments that might trigger new friendships or conflicts within a group. By working closely with diverse groups of young people and using different media, we observed how the latter influenced interactions and the atmosphere within each group. The digital participation group explored new territory online, embracing trial and error as we created an entirely new space with a technology of which none of us (other than our civic tech partner) had prior experience. This shared ignorance created space for cheerful experimentation (see also Börner et al., 2023). A lack of knowledge of what might be expected of a digital participation process also allowed the young people to use the various components offered within the platform in original ways, reinventing meanings of participation and pushing the boundaries of how the platform could be used (Bussu et al., 2024).

I’ve been enjoying it, yes […] Changing mental health and also creating new ideas. How to get young people to interact [online], especially people of colour. (Digital Peer Designer, June 2023)

I think it’s been so good that we’ve been able to do that because we’ve all been able to share our ideas. If I have an idea, and everyone else shares their opinions and then implement it all, something like everyone’s had an equal kind of role to implement what they want into it which was nice. (Digital Peer Designer, June 2023)

By contrast, the research group brought with them expectations of what research meant based on previous encounters with “trained researchers”, teachers and lecturers. This initially affected their own perceived (lack of) expertise vis-à-vis academics and generated a degree of risk aversion to embracing more explorative and imaginative approaches.

At the start it was hard to keep [your feet] on the ground at times. It’s kind of like little turtles running around the beach. (Peer Researcher, May 2023)

Informal conversations, serendipitous exchanges and connections within the group, particularly on shared experiences of the impact of different cultural backgrounds on their mental health, helped them redefine their own roles as evaluators of the project and shape their own tools of data collection. This was a slow process that took several months of weekly meetings where some people felt at times unsure of what the meetings were about.

Within the project itself, the looser structure meant less clarity on division of work and responsibilities and really highlighted the importance of communication for this work – it was an issue for some of us. We worked through weekly emails and a WhatsApp group alongside our weekly in-person meetings, but this comms wasn’t always easily accessible for everyone, which meant that response times varied and communication was a challenge. (Extract from peer researcher’s blog, May 2023)

**Figure 6.**
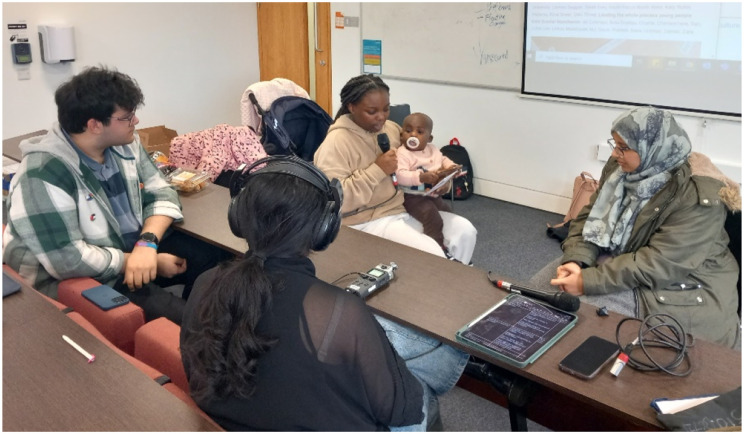
Improvised podcasting studio for Mindset Revolution

The turning point was the realisation that podcasts (Figure 6) could be used as a research tool to explore their experience in a safe and anonymous space. This also triggered greater confidence within the group, with different people starting to use things they felt they were good at – for example, spoken-word poetry, drawings, or writing blogposts - as research methods.

Working on that podcast has led to some valuable discussions (which were highlighted by many members of the research group as one of the more valued aspects of the work, allowing for learning from and connecting with people of different experiences) and some developing skills – the editing, scripting, and recording was all done by us. (Extract from peer researcher’s blog, May 2023)

In the evaluation playbook that brings together their reflections on the project (Ahmed et al., 2023, p.30), the peer researchers finally concluded:

**Anything can be research**. Mental health research can be a bit clinical and scientific and needs to make space for the lived experience of young people within democratic processes to ensure there is meaningful change.

As McFarlane (2011b) puts it, actors and processes are (re)defined by the assemblages they enter and reconstitute, emphasising the potentiality of sites and actors and their capacity to be more than the sum of their connections.

It is not a novel device that opens possibilities of change but the novelty of the arrangements with other objects and activities (McFarlane, 2011a). This process of socio-material reassembling can transform old spaces and materials through new uses, challenging power hierarchies in subtle ways: lecture theatres and computer rooms were slowly reclaimed as informal community spaces with food and drinks, where to improvise new plays, connect via hybrid (digital/analogue) sessions for brainstorming, or reimagine data through colourful jigsaws and poems (see extract below).

Late Legislation

The theatre of your life will come into law

So you don’t have to fight constantly against the bourgeois

Liberated from the shackles of institution

Don’t you worry my dear you’ll get your restitution

(Extract from a poem by a peer researcher reflecting on one of the Legislative Theatre rehearsal sessions, March 2023)

This process interacts with and challenges pre-existing conceptualisations of those spaces and the hierarchies associated to them, reflecting evolving aspirations and relationships.

We’ve provided a space for young people such as ourselves to sit and ultimately, play and look at the way certain institutions or just any, any external spaces and places that they reside in, how […] that certain space that they reside in the world, how that plays a part in the daily wellbeing or the lack thereof. In doing so, so that we could all come together and try to kinda like, lift, not lift each other. Yeah, be like that caring pat on the back and be like ‘hey’ we see what you’re seen. (Legislative Theatre, peer facilitator, June 2023)

Including youth in the design process did not automatically lead to equal relationships, particularly as we were working with young people dealing with complex personal struggles. Different and contingent forms of power inevitably highlight sociocultural and material exclusions. Despite reclaiming academic spaces, existing hierarchies often undermined young people’s ownership of these areas. For example, security staff sometimes restricted access until booking were confirmed or monitored the young people near closing time to ensure they were leaving. Although young people transformed the space during sessions, these interactions reminded them that it was not truly theirs, and the power to deny access was ever-present.

Power dynamics influenced the process from the outset. We recruited a very diverse group of young people, along gender, race and class lines, but selecting representatives based on adult-defined diversity can be problematic (Grant-Smith & Edwards, 2011, p. 13). O’Donoghue et al. (2002, p. 21) suggest that to support effective participation, we should move beyond representation and recognise young people’s rights to participate as individuals, allowing them to self-(re)define their identities through participation.

I feel like when we come together as a research group, we kind of all shared similar experiences, whether that be from having an intersectional background, being queer being Black, being global majority and I feel that’s really informed a lot of the work that we have done, what we’re continuing to try and shape […]. (Peer researcher, June 2023)

There is often an expectation that young people should think, behave, and engage like adults (Camino & Zeldin, 2002, p. 215). However, as Grant-Smith and Edwards (2011, p. 112) note “equality of participation does not imply that all participants enter the participatory arena with an equal capacity for participation”. Ignoring these differences can worsen inequalities. Genuine co-creation occurs only when young people see themselves as equal partners and perceive their relationship with adults as balanced (Bowler et al., 2021; see also Börner et al., 2021). For example, initially, young people asked permission to speak or act, reproducing classroom dynamics. Acknowledgment of these power imbalances was crucial and involved tackling issues of positionality, ethics, influence, and relational skills, through ongoing reflective practice. Mutual trust is a vital component to address power imbalances and enable honest dialogue and reflectivity, but building trust takes time, and time is often lacking in short funding cycles and action research micro-projects.

**Figure 7.**
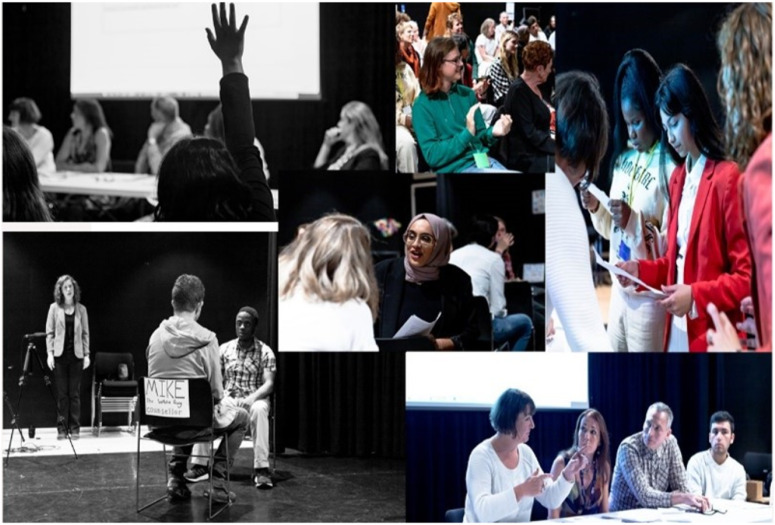
Legislative theatre performance, Optimistic Minds, September 2022

The group of four who trained as LT facilitators perhaps went farthest in recasting themselves in new roles vis-à-vis the adults in the project. These young people had been involved in our previous project (see Figure 7) and already knew each other and many of the adults in the team. Their story of success also highlights the importance of time to build trust within a group of strangers.

To make the play, we start with games and discussions. We’re learning how to teach the rules of the games, but another thing we’re learning is how to discuss the games and think about what they’re leading a group to understand. Like, this game is about destabilizing people so that they can imagine things differently, while this game is about revealing ingrained rules in our world. It’s interesting how something that seems like a simple game – if you really think about it and engage with it, the game can yield interesting results. I’ve been realising how conscious you have to be about what you’re saying: so that you’re including everyone, going through the process with them rather than doing it to them. (Extract from LT peer facilitators’ blog, March 2023)

The quote above also helps unpack a crucial aspect of LT as a practice that is based on structured and purposeful fun to dismantle power dynamics and co-create new relational dynamics, through openness to discovery and play. Arts-based practice helped disrupt and reconfigure social spaces, challenging dominant ways of seeing and engaging with the world (Beyes & Steyaert, 2011; Cunningham et al., 2024). Young people could make noise, take space, act silly, while considering seriously what social change can mean and what it should look like.

This new agency, however, at times pushed the young actors beyond their comfort zone, in a way that was as empowering as it was daunting, as highlighted by two LT peer facilitators.

When I think about doing this Legislative Theatre facilitation thing in public I feel worried, because sometimes I don’t always want to talk - to be public. Sometimes I feel like a tree…. That just exists, listening, silently. So I’ve been wondering, can I be a tree, or do I need to talk as the facilitator? But then I remember, I’m not here to deliver knowledge. Actually, I can listen, I can facilitate and still be a tree. It would be different if you were standing up at the front and saying, “This is how the play will go.” But that’s not what we’re doing. The people in the group have a lot of freedom to create whatever they want, without the facilitator leading it. So I can speak, but I don’t have to give the answers. (LT peer facilitator, March 2023)

It feels like we’re getting to see behind the scenes, where you usually don’t get to see in a community project. A lot of work goes into facilitation! […] Like any creative process, you see someone who’s been doing it for ages and you think they’re really talented, but actually they’ve been working really hard and practicing for a long time. Which makes facilitation feel daunting but also more possible: this is something we can also learn to be good at! (LT peer facilitator, March 2023)

Within these socio-material assemblages, non-human components, from transport and academic strikes to malfunctioning podcast equipment or undelivered snacks at some meetings, combined with human participants’ (both the adults and the young people) personal fragilities and complex mix of personal problems and career ambitions. These dynamics continuously put at peril precarious relationships of trust. As noted by Durose et al. (2022, p. 2140), “[labour of] assembling is not only about bringing socio-material resources together but also about developing and nurturing them in particular contexts or places.” This intensive work on the ground often happens without an adequate institutional infrastructure that can support it.

### Embedding change through generating and sustaining commitment and action

Embeddedness of a participatory culture requires integration and sustained presence of participatory practices within the broader institutional and social context. It involves ensuring that participatory mechanisms are not just temporary or superficial but deeply rooted in practical dimensions of governance, making them a fundamental part of decision-making and policy implementation (Bussu et al., 2022). Our participatory assemblage was inevitably shaped by existing structures of power and capital underpinning policy and academia, often in conflict with the participatory and transformative aims and ambitions of our action research project. It was moulded by different discourses, at times at odds with each other (i.e., participatory research, medical policy and practice, technology). It also included very different actors: diverse young people studying, working or unemployed, academics from different disciplinary backgrounds, third sector partners, artists, mental health professionals, policymakers, funders; all with different ways of working, at times with diverging priorities.

Young people were supported to flip traditional public consultation processes, and policymakers were invited to youth-led spaces as respondents to youth proposals and as contributors to the youth-led deliberation during LT performances or online dialogues. This process required developing and mobilising new methods and perspectives (Durose et al., 2022). The adult partners acted as gatekeepers, connecting policymakers (e.g., health practitioners, GMCA health policymakers, parliamentary ombudsman, third sector organisations) to these novel spaces. However, policymakers at times met these invitations with scepticism due to their accustomed roles of controlling agendas and outcomes. Partners also sought to collaborate with other grassroots and youth groups, but competition for funding and visibility sometimes hindered cooperation. The importance of creating and maintaining a “buffer zone” (Bennett & Brunner, 2022), a border zone for fostering complex collaborations across diverse epistemic worlds, was recognised by all, and yet it was inevitably challenging to nurture it:

We could have probably brought policymakers on to the steering group and that might have got some extra buy in and commitment on that side as well (Partner, July 2023)

But all of that [reaching out to policymakers] takes a lot of time and capacity and kind of you have to knock on their door over and over, and you have to put a lot of pressure on them, and you have to. You know, you have to have the sort of time and space to put that pressure on them. (Partner, July 2023)

Whereas there is an important role for individuals in sustaining these connections through labour of assembling, limited material resources and different priorities become inevitable constraints. The literature on action research recognises that “transformative aspirations and change processes inevitably come with ambiguities, mistakes, frustrations, tensions, conflicts and disappointments” (Bartels & Friedman, 2022, p. 99). By focusing only on the positive aspects of ART, we risk creating unrealistic expectations that can lead to frustration and cynicism. Instead, a balanced approach that acknowledges and addresses challenges through critical reflexivity and honest dialogue is paramount (ibid.). We held regular meetings to nurture a shared vision and identify and address conflictual dynamics, but not all partners were able to attend regularly, and disagreement on project ambitions and underlying aims was not always recognised, and thus went unresolved in some instances.

Some policymakers, including from mental health services and local authorities, and who had already committed to specific policy actions, continued to be involved, providing updates on progress. Others stopped responding to our invitations. Non-linear and oftentimes delayed impacts generated feelings of deflation among some of the young people.

I think it would have been nice to have more contact with like policymakers throughout the process […] Given the fact that it was youth led it came from a position of [lived] experience and opposed to people speaking on our behalf. So it could really get our message across by being such a youth led project. (Peer digital designer, June 2023)

It takes time and ongoing relational labour of assembling to build a rapport of trust, not only within a partnership and between adults and the young people, but also with policymakers. Partners observed that policymakers were often apprehensive about entering unfamiliar spaces and reluctant to acknowledge their influence on policy changes, “my role is only X, there’s not much I can change”. More time and resources might help strengthen these relationships, encouraging policymakers to embrace a more participatory approach in their day-to-day work. Policymaking is complex and involves constant shifts in priorities, which can make it challenging to maintain focus and institutional memory, especially over long-term commitments.

Through an assemblage lens, agency for impact is understood as a distributed force that emerges from interaction between diverse actors (e.g., participants, partners, the wider community, as well as policymakers) and different elements (e.g., institutional and bottom-linked spaces, digital and in person participation, personal and professional relationships) (cf. Durose et al., 2022). This perspective thus better recognises the agency of the young people and their communities on policy change. While young people were sometimes frustrated by the lacklustre responses from policymakers, they also began to assert their right to accountability and follow-up:

I think one thing that we definitely kind of learned when dealing with policymakers is that people don't want to claim the power that they have. And that's like a really awful thing for like most people because we all do have, like, power, especially when we come together, and us not realizing that is kind of how systems perpetuate [oppressions]. But also when people get to positions of power they like refuse to own up to that and they refuse to like, you know, acknowledge that and kind of trying to use that for good things. This [policymaker] who was on the panel [at the LT play] and kept saying stuff like, oh, well, I’m only on this committee, so I can't put these things in place. And it’s like you’re on the committee. You’ve got so much more power than like any of us have, like […]. So it’s kind of I guess, like learning but like actually, like I’m, I do have like a right to hold people accountable. And I do have like a right to kind of get angry at people, if that’s what’s going to be transformative. (LT peer facilitator, June 2023)

Assemblage acknowledges contingency, where impact is emergent and non-linear. This stance encourages action researchers and participatory democrats to move beyond ad hoc events ending with a list of recommendations to be implemented. It supports open-ended partnerships between researchers, citizens, civil society and local institutions to co-create strategies to navigate the complexities of implementation, placing emphasis on collective oversight and scrutiny (Bussu et al., 2024). In this respect, funders also have a responsibility to ensure that programmes include the building of institutional leadership to support continuity beyond the confines of short funding cycles (Patel, 2022, p. 386).

At the time of writing, some of the partners continue to support different strands of the project and new alliances are being built with sympathetic policymakers, whose agenda is slowly (often serendipitously) aligning with some of the young people’s policy ideas, for instance on youth-informed training for mental health staff.

What I’m hoping is that this is an achievement that continues on for next generations of young people. […] What I can say is that we are committed to whatever the young people are saying they want to do next in terms of actions. (Partner, June 2023)

Crucial to this work is the transformation of young people’s lives, with emphasis on diversity, interconnectedness and inclusivity. The practices we adopted were shaped by these young people and the constraints and opportunities of their lives outside the project. The openness and flexibility of our approach prioritised inclusivity and participants’ needs over a detailed roadmap and targets.

I think I enjoyed that we didn’t need to be perfect. That, that really made a massive difference. Like we didn’t need to be perfect and that kind of made it perfect. You know, we… we came together with all sorts of ideas and, and plans and some work some didn’t. But like we still managed to create all of this. So I think that was the best part. You know, we were allowed to be human during this project. [Peer researcher, July 2023)

Many of the young people continue to be involved in the MR collective through new funding for related activities. Some young people are now working on other LT initiatives with local institutions, as well as partners’ new projects on youth mental health or other youth policies. This is an important measure of embeddedness, as this participatory assemblage reassembles into new ones.

## Conclusion

This paper presented a novel approach to youth participatory democracy and action research for social transformation on mental health, where young people co-created multiple arts-based and digital spaces and evaluated their own experience of participation and social impact. We build on existing work on arts-based (Beyes & Steyaert, 2011) and youth ART (Börner et al., 2021, 2023; Bowler et al., 2021; Flicker et al., 2008; Luguetti et al., 2024), and we contribute to expanding current practice and analysis in two ways.

Firstly, MR’s youth-led approach was grounded in the assumption that transformative change can only happen if it is owned and shaped by groups that are marginalised in current policy and research environments, such as young people, and if it is embedded in their own lives and social contexts. However, power imbalances are always present. At different points we acknowledged relations of power between the young people, the adults in the room and the other adults (individuals and organisations) in the project who were not always in the room but might be named and have an influence – for example, employers, funders or tech support. The disruptive creativity and playfulness of LT and the digital platform helped the young people recognise and challenge power hierarchies through joyful practice. The participatory evaluation embedded in the project from the very beginning informed ongoing reflectivity that shed light on power imbalances and conflicts, from the young people’s perspective. A flexible and adaptable approach based on radical inclusivity progressively helped enhance rather than extract the young participants’ own resources and agency. This agency finally translated into a strong focus on intersectionality in youth mental health support, reflecting the experiences of a very diverse group of young people, whose intersectional experiences of oppression are often silenced in the health system or educational settings.

Secondly, the paper used assemblage theory to provide an original and fine-grained analysis of the multiple human and material components that interacted to shape a participatory project such as MR, showing how these interactions developed to open or close space for youth-led action. Assemblage components arrange differently and develop different capacities across different contexts. In this respect, an assemblage perspective warns against blueprints and top-down participatory designs that envisage ideal, but not necessarily achievable, synergies (Bussu et al., forthcoming). Instead, it encourages situated and relational practices. The paper thus recognises the vital importance of ongoing labour of assembling in driving and sustaining relational processes that can lead to transformative change, while also acknowledging complex power dynamics that continuously disrupt and challenge precarious buffer zones.

An assemblage-informed analysis recognises power as a distributed force, where young people and their community also have agency on policy change and should be supported in demanding follow up from policymakers. This entails that a participatory process such as MR cannot stop at recommendations but might need to encourage more collective scrutiny, advocacy and campaigning to foster and energise a sustainable youth movement for mental health that works with and beyond state institutions to affect social change (Bussu et al., 2024). The assemblage lens highlights how any ART projects need to continuously adapt to emerging opportunities to identify synergies with policy agendas and grassroots action for long-term sustainability. Within an ART assemblage, action researchers and practitioners are pivotal intermediaries, creating connections and building alliances across different epistemic worlds and nurturing new spaces of action for marginalised voices to shape their own visions for change.

Further research should explore strategies and methods, as well as socio-economic and political constraints, to reimagine ART as a participatory ecology for social change, driven by marginalised and seldom heard groups. This may involve redefining the roles of ART researchers and practitioners, moving away from ad hoc projects led by individuals and small partnerships towards collectives of activist-researchers and practitioners embedded in a given community (geographical, of interest, or practice) to build cross-sectoral constituencies of change and at different scales.

## Notes

1 Funded by UKRI/ RSA.

2 The adult partners included three academics with expertise in participatory democracy, coproduction and youth mental health; Manchester-based charities working on youth participation and arts-based and creative methods to strengthen youth mental health; one legislative theatre practitioner; a Barcelona-based foundation designing and supporting civic tech and digital participation based in Spain; and a Manchester-based community radio.

3 The proposals reflect this diverse group’s concerns with institutional racism and cultural barriers to mental health support.

4 The playbook can be accessed here https://openspaces.platoniq.net/processes/mindset-revolution-resources?locale=en.

## References

[bibr44-14767503251320616] Ahmed M. Saira A. Changwereza M. J. , et al. (2023) Mindset Revolution: youth voice on mental health: what we learnt. Available at: https://openspaces.platoniq.net/processes/mindset-revolution-resources/f/413/

[bibr1-14767503251320616] Allahwala A. Bhatia A. (2022). Supporting youth-led community geography on the impacts of neighbourhood social infrastructure on young people’s lives: A case study from East Scarborough, Canada. Geojournal, 87(S2), 329–342. 10.1007/s10708-021-10473-8

[bibr2-14767503251320616] Austin S. L. (2010). Children’s participation in citizenship and governance. In Percy-Smith B. Thomas N. (Eds.), A handbook of children and young people’s participation (pp. 245–253). Routledge.

[bibr3-14767503251320616] Bartels K. P. R. Friedman V. J. (2022). Shining light on the dark side of action research: Power, relationality and transformation. Action Research, 20(2), 99–104. 10.1177/14767503221098033

[bibr4-14767503251320616] Bennett H. Brunner R. (2022). Political and ethical dilemmas in multi-agency participatory research: The role of the buffer zone. Methodological Innovations, 15(3), 387–399. 10.1177/20597991221129775

[bibr5-14767503251320616] Beyes T. Steyaert C. (2011). The ontological politics of artistic interventions: Implications for performing action research. Action Research, 9(1), 100–115. 10.1177/1476750310396944

[bibr6-14767503251320616] Boal A. (1998). Legislative theatre: Using performance to make politics. Routledge.

[bibr7-14767503251320616] Börner S. Kraftl P. Giatti L. L. (2021). Blurring the ‘-ism’ in youth climate crisis activism: Everyday agency and practices of marginalized youth in the Brazilian urban periphery. Children's Geographies, 19(3), 275–283. 10.1080/14733285.2020.1818057

[bibr8-14767503251320616] Börner S. Kraftl P. Giatti L. L. (2023). More than participatory? From ‘compensatory’ towards ‘expressive’ remote practices using digital technologies. Qualitative Research, 24(3), 459–485. 10.1177/14687941231165882

[bibr9-14767503251320616] Bowler L. Wang K. Lopatovska I. Rosin M. (2021). The meaning of “participation” in Co-design with children and youth: Relationships, roles, and interactions. Proceedings of the Association for Information Science and Technology, 58(1), 13–24. 10.1002/pra2.432

[bibr10-14767503251320616] Bradbury H. (2022). How to do action research for transformations. Edward Elgar.

[bibr11-14767503251320616] Bradbury H. (2024). Methodology for a time of eco-social planetary crisis: Action research helping transformations happen. Action Research, 22(2), 107–113. 10.1177/14767503241255488

[bibr12-14767503251320616] Bradbury H. Waddell S. O’ Brien K. Apgar M. Teehankee B. Fazey I. (2019). A call to Action Research for Transformations: The times demand it. Action Research, 17(1), 3–10. 10.1177/1476750319829633

[bibr13-14767503251320616] Bussu S. Bua A. Dean R. Smith G. (2022). Introduction: Embedding participatory governance. Critical Policy Studies, 16(2), 133–145. 10.1080/19460171.2022.2053179

[bibr14-14767503251320616] Bussu S. Senabre Hidalgo E. Schulbaum O. Eve Z. (2024). Make (digital) space for and with the young: Arts-inspired co-design of civic tech for youth mental health policies. Convergence. OnlineFirst. 10.1177/13548565241264001

[bibr15-14767503251320616] Bussu S. Wojciechowska M. Dias T. Forde C. (forthcoming). Participation as Assemblage- Looking at developments in democratic innovations through an assemblage lens. Politics.

[bibr16-14767503251320616] Bustamante Duarte A. M. Degbelo A. Kray C. (2018). Exploring forced migrants (Re)settlement & the role of digital services. In Proceedings of 16th European conference on computer-supported cooperative work - exploratory papers, reports of the European society for socially embedded technologies (Vol. 2, pp. 1–18). European Society for Socially Embedded Technologies (EUSSET).

[bibr17-14767503251320616] Camino L. Zeldin S. (2002). From periphery to center: Pathways for youth civic engagement in the day-to-day life of communities. Applied Developmental Science, 6(4), 213–220. 10.1207/s1532480xads0604_8

[bibr18-14767503251320616] Cunningham M. Rubin K. Woods S. (2024). People aren’t going to show up to the revolution if it isn’t fun’ theatre of the oppressed and disrupting the tone of voice within deliberative democracy. In Weston S. (Ed.), Applied theatre: Voice - performance and social justice (pp. 95–114). Bloomsbury Publishing.

[bibr19-14767503251320616] DeLanda M. (2016). Assemblage theory. Edinburgh University Press.

[bibr20-14767503251320616] Deleuze G. Guattari F. (1988). A thousand plateaus: Capitalism and schizophrenia. Bloomsbury.

[bibr21-14767503251320616] Dewey J. (1927). The public and its problems.

[bibr22-14767503251320616] Durose C. van Ostaijen M. van Hulst M. Escobar O. Agger A. (2022). Working the urban assemblage: A transnational study of transforming practices. Urban Studies, 59(10), 2129–2146. 10.1177/00420980211031431

[bibr23-14767503251320616] Flicker S. Maley O. Ridgley A. Biscope S. Lombardo C. Skinner H. A. (2008). e-PAR: Using technology and participatory action research to engage youth in health promotion. Action Research, 6(3), 285–303. 10.1177/1476750307083711

[bibr24-14767503251320616] Freire P. (1968). Pedagogy of the oppressed. Seabury Press.

[bibr25-14767503251320616] Grant-Smith D. Edwards P. B. (2011). It takes more than good intentions: Institutional and attitudinal impediments to engaging young people in participatory planning, Journal of Public Deliberation, 7(1), Article 11. 10.16997/jdd.122

[bibr26-14767503251320616] Grasso M. T. Farrall S. Gray E. Hay C. Jennings W. (2018). Socialization and generational political trajectories: An age, period and cohort analysis of political participation in Britain. Journal of Elections, Public Opinion, and Parties, 29(2), 199–221. 10.1080/17457289.2018.1476359

[bibr27-14767503251320616] Herbert S. (2000). For ethnography. Progress in Human Geography, 24(4), 550–568. 10.1191/030913200100189102

[bibr28-14767503251320616] Horton J. Kraftl P. (2009). Small acts, kind words and ‘not too much fuss’: Implicit activisms. Emotion, Space and Society, 2(1), 14–23. 10.1016/j.emospa.2009.05.003

[bibr29-14767503251320616] Kallio K. P. Häkli J. Bäcklund P. (2015). Lived citizenship as the locus of political agency in participatory policy. Citizenship Studies, 19(1), 101–119. 10.1080/13621025.2014.982447

[bibr30-14767503251320616] Luguetti C. Ryan J. Eckersley B. Howard A. Craig S. Brown C. (2024). “Everybody’s talking about doing co-design, but to really truly genuinely authentically do it […] it’s bloody hard”: Radical openness in youth participatory action research. Action Research, 22(4), 307–326. 10.1177/14767503231200982

[bibr31-14767503251320616] Lundy L. O’Donnell A. (2021). Partnering for child participation: Reflections from a policy-maker and a professor. In Horgan D. Kennan D. (Eds.), Child and youth participation in policy, practice and research (pp. 15–21). Routledge.

[bibr32-14767503251320616] Massey D. B. (2005). For space. Sage.

[bibr34-14767503251320616] McFarlane C. (2011a). Assemblage and critical urbanism. City, 15(2), 204–224. 10.1080/13604813.2011.568715

[bibr35-14767503251320616] McFarlane C. (2011b). The city as assemblage: Dwelling and urban space. Environment and Planning D: Society and Space, 29(4), 649–671. 10.1068/d4710

[bibr36-14767503251320616] Nishiyama K. (2017). Deliberators, not future citizens: Children in democracy, Journal of Public Deliberation, 13(1), Article 1. 10.16997/jdd.267

[bibr37-14767503251320616] O’Donoghue J. L. Kirshner B. McLaughlin M. (2002). Introduction: Moving youth participation forward. New Directions for Youth Development, 96, 15–26. 10.1002/yd.24 12630271

[bibr38-14767503251320616] Patel Z. (2022). The potential and pitfalls of co-producing urban knowledge: Rethinking spaces of engagement. Methodological Innovations, 15(3), 374–386. 10.1177/20597991221129779

[bibr39-14767503251320616] Pateman C. (1970). Participation and democratic theory. Cambridge University Press. 10.1017/CBO9780511720444

[bibr40-14767503251320616] Percy-Smith B. Cuconato M. Reutlinger C. Thomas N. P. (2019). Action research with young people: possibilities and ‘messy realities’, Diskurs Kindheits- und Jugendforschung / Discourse. Journal of Childhood and Adolescence Research, 3, 255–270. 10.3224/diskurs.v14i3.02

[bibr41-14767503251320616] Ritterbusch A. E. Boothby N. Mugumya F. Wanican J. Bangirana C. Nyende N. Ampumuza D. Apota J. Mbabazi C. Nabukenya C. Kayongo A. Ssembatya F. Meyer S. R. (2020). Pushing the limits of child participation in research: Reflections from a youth-driven participatory action research (YPAR) initiative in Uganda. International Journal of Qualitative Methods, 19(1), 160940692095896. 10.1177/1609406920958962

[bibr42-14767503251320616] Shore C. Wright S. (1997). Anthropology of public policy: Critical perspectives on governance and power. In Shore C. Wright S. (Eds.), Policy: A new field of anthropology (pp. 201–216). Routledge.

[bibr43-14767503251320616] Thévenot L. (2014). Engaging in the politics of participative art in practice. In Zembylas T. (Ed.), Artistic practices: Social interactions and cultural dynamics (pp. 132–150). Routledge.

